# Impact of renal complications on outcome in adult patients with acute fulminant myocarditis receiving venoarterial extracorporeal membrane oxygenation: an analysis of nationwide CSECLS database in China

**DOI:** 10.1186/s13613-023-01186-x

**Published:** 2023-09-27

**Authors:** Tong Hao, Lei Chen, Changde Wu, Jianfeng Xie, Chenglong Li, Haixiu Xie, Zhongtao Du, Ling Liu, Yi Yang, Songqiao Liu, Xiaotong Hou, Haibo Qiu

**Affiliations:** 1 Jiangsu Provincial Key Laboratory of Critical Care Medicine, Department of Critical Care Medicine, Trauma Center, Zhongda Hospital, School of Medicine, Southeast University, Nanjing, 210009 Jiangsu People’s Republic of China; 2 Center for Cardiac Intensive Care, Beijing Anzhen Hospital, Capital Medical University, Beijing, People’s Republic of China; 3 Nanjing Lishui People’s Hospital, Zhongda Hospital Lishui Branch, Southeast University, No. 86 Chongwen Road, Lishui District, Nanjing, 211200 Jiangsu People’s Republic of China

**Keywords:** Extracorporeal membrane oxygenation, Renal complications, Risk factors, Mortality

## Abstract

**Background:**

Limited data are available on renal complications in patients with acute fulminant myocarditis (AFM) receiving venoarterial extracorporeal membrane oxygenation (VA-ECMO) support in China. To evaluate the impact of renal complications on outcomes in adult patients with AFM supported with VA-ECMO.

**Methods:**

Data were extracted from Chinese Society of ExtraCorporeal Life Support (CSECLS) Registry database. Adult patients who were diagnosed with AFM receiving VA-ECMO support in the database were included. The primary outcome was 30-day mortality in patients with AFM supported with VA-ECMO. Logistic regression model was used to examine the impact of renal complications on 30-day mortality by adjusting confounders.

**Results:**

A total of 202 patients were included. The median age was 38 years (IQR 29–48) and males (*n* = 103) represented 51.0% of the total accounted patients. The median ECMO duration was 142.9 h (IQR 112.1–188.8 h). 178 (88.1%) patients weaned from ECMO and 156 (71.9%) patients survived. 94(46.5%) patients developed renal complications while on ECMO course. Patients with renal complications had higher 30-day mortality (40.7% (37 of 94) vs 8.3% (9 of 108), *P* < 0.001) compared with those without. The development of renal complications was related to a 3.12-fold increase risk of 30-day mortality (adjusted OR 3.120, 95%CI 1.002–6.577, *P* = 0.049). Increasing age (adjusted OR1.025, 95% CI 1.008–1.298, *P* = 0.040) and higher SOFA score (adjusted OR 1.162, 95%CI 1.012–1.334, *P* = 0.034) were independent risk factors of renal complications.

**Conclusions:**

Our findings demonstrated that patients with AFM receiving VA-ECMO at high risk of developing renal complications. Advancing age and higher SOFA score was associated with increased risk of developing renal complications. The onset of renal complications was significantly associated with 30-day mortality.

**Supplementary Information:**

The online version contains supplementary material available at 10.1186/s13613-023-01186-x.

## Introduction

Acute myocarditis presents with heterogeneous signs and symptoms ranging from subclinical disease to chest pain that can mimic myocardial infarction or pericarditis, refractory cardiogenic shock, or sudden cardiac death from ventricular fibrillation [1, 2]. Acute fulminant myocarditis (AFM) is characterized by rapid onset of cardiogenetic shock necessitating inotropic drugs and/or mechanical circulatory support, corresponding to 30% hospital patients of acute myocarditis [1, 3]. Mechanical circulatory supports including extracorporeal membrane oxygenation (ECMO) have been widely used to support patients with circulatory shock as a bridge to cardiac recovery or decision because of improved ECMO-related technology and promptness of applications [4–6].

Early organ dysfunction may have an impact on a patient's prognosis and quality of life. The complex nature of single organ failure potentially leading to multiple organ dysfunction syndrome in critically ill patients due to organ cross-talk, which is a crucial aspect of human biology [7]. Rather than a primary disease, acute kidney failure is considered a window to a potentially serious underlying systemic disease. Renal complications are infrequent among patients receiving ECMO support, with an occurrence rate ranging from 26 to 85% [8–10]. Renal complications are more common in venoarterial extracorporeal membrane oxygenation (VA-ECMO) than in venovenous extracorporeal membrane oxygenation (VV-ECMO) resulting in adverse outcome [11, 12]. The underlying mechanisms for renal complications among patients requiring VA-ECMO are extremely complex. Various factors including premorbid conditions [13], primary underlying disease (e.g., hemodynamic instabilities, inflammatory responses and immune-mediated injury) [13–15] and the ECMO circuit [16] predispose patients to incident or exacerbation of renal complications.

ECMO have been used in patients with AFM for many years in China [17]. Some small-scale studies have reported the incidence rate and (or) risk factors of renal complications developing while on VA-ECMO [18–21]. By contrast, there are only scarce data on the effect of renal complications on mortality of patients with VA-ECMO support [10, 22]. To inform clinical practice and design future studies for prevention and management of this high-risk group, understanding the impact of renal complications and its contributing factors is essential. Hence, we conducted this retrospective observational national-level study aiming to investigate the incidence rate of renal complications and assess the impact on outcome of renal complications developing in patients with AFM receiving VA-ECMO support in China.

## Methods

### Study design and setting

We conducted a retrospective observational study using electronic health records data from the Chinese Society of ExtraCorporeal Life Support (CSECLS) Registry database (ClinicalTrials.gov registration number: NCT04158479). The CSECLS registry, as a voluntary registry, collects information on the use, complications, and outcomes following ECMO support in adults and children from 112 pediatric and adult ECMO centers in China. Data were collected using a standardized electronic reporting sheet, submitted via the organization’s website. Accuracy is augmented by a point-of-entry data assessment with error and validity checks and database managers. One author (S.Liu) obtained access to the database and was responsible for data extraction. Approval from the central Institutional Review Board of Beijing Anzhen Hospital (2019040X) was obtained. Our study complied with the STrengthening the Reporting of OBservational studies in Epidemiology (STROBE) statement (Additional file 1).

From January 1, 2017 to December 31, 2019, all adult patients with AFM received VA ECMO therapy reported to CSECLS Registry database were included. The diagnosis of AFM was based on the guideline of European Society of Cardiology (Additional file 2: Table S1) [1, 23]. The exclusion criteria were as follows: (1) chronic kidney disease, is defined as abnormalities of kidney structure or function shown by glomerular filtration rate (GFR) of less than 60 mL/min per 1.73 m^2^, or markers of kidney damage, or both, at least 3 months duration according to Kidney Disease: Improving Global Outcomes (KDIGO) 2012 Clinical Practice Guideline; (2) duration of ECMO support was less than 48 h [24]. Patients who met inclusion criteria multiple times in different ICU stays within a year were included only once. Cases beyond 31 December 2019 were not included because the SARS–CoV-2 pandemic may have affected the provision of pre-hospital and intensive care services.

## Data collection

Baseline characteristics including demographic information, comorbidities, main diagnose at admission, severity of illness assessed by Sequential Organ Failure Assessment (SOFA) score [25], survival after veno-arterial-ECMO (SAVE) score [26] were collected.

The following data were recorded prior to ECMO initiation: ECMO indication (circulatory failure or extracorporeal cardiopulmonary resuscitation (ECPR)), cardiac arrest, blood pressure, arterial blood gas, ejection fraction (EF), organ support excluded ECMO, maximal doses of vasopressors and vasoactive-inotropic score (VIS). VIS was calculated using the maximum doses of vasoactive and inotropic drugs (VIS = dopamine dose [μg kg-1 min^−1^] + dobutamine [μg kg-1 min^−1^] + 100 × epinephrine dose [μg kg^−1^ min^−1^] + 50 × levosimendan dose [μg kg^−1^ min^−1^] + 10 × milrinone dose [μg kg^−1^ min^−1^] + 10,000 × vasopressin [units kg^−1^ min^−1^] + 100 × norepinephrine dose [μg kg^−1^ min^−1^]) [27].

Within 24 h after ECMO initiation, we collected clinical data including blood flow and arterial blood gas, SOFA score, VIS, other mechanical circulatory support. The duration of ECMO support and invasive mechanical ventilation (IMV) and length of hospital and intensive care unit (ICU) stay were also collected.

## Definition

Renal complications were defined by change in creatinine or requirement for renal replacement therapy (RRT) according to data definitions from Extracorporeal Life Support Organization (ELSO). Specifically, renal complications were defined as patients newly acquired a serum creatinine level greater than 1.5 mg/dl or requirement of RRT (including peritoneal dialysis, continuous venovenous hemodiafiltration, continuous venovenous hemofiltration or hemodialysis) after ECMO initiation [28, 29].

## Outcomes

The primary outcome was the 30-day mortality after ECMO initiation in patients with AFM. We also assessed the 90-day mortality after ECMO initiation, incidence rate and risk factors of renal complications.

## Statistical analysis

Values are presented as the median (interquartile range [IQR]) or mean (standard deviation) for continuous variables as appropriate and as the total number (percentage) for categorical variables. Comparisons between groups were made using the Kolmogorov–Smirnov test or Student’s t test for continuous variables and chi-square or Fisher’s exact test for categorical variables. Logistic regression was used to assess the impact of renal complications on likelihood for 30-day mortality by adjusting other prognostic factors and to identify risk factors of renal complications.

Risk factors for the 30-day mortality included baseline characteristics (age, severity of illness, comorbidities), and variables prior to ECMO initiation and during ECMO course at 24 h after ECMO. Severity of illness before ECMO initiation was assessed by SOFA score. Candidate risk factors associated (*P* < 0.1) with this outcome in the univariable analysis and with missing values less than 20% were introduced into the multivariate model, and the final model was selected using stepwise selection method.

Risk factors for the renal complications were baseline characteristics (age, severity of illness, comorbidities) and variables prior to VA-ECMO with regard to the onset time of renal complications. Regarding the secondary outcome, the final multivariable logistic regression model was obtained using the same selection method as used for the analysis of the primary outcome. The discrimination abilities were quantified by the area under the receiver operating curve (AUROC). Goodness of fit was verified by the Hosmer–Lemeshow test. Cumulative survival curves for 30 days follow-up was generated utilizing the Kaplan–Meier method and compared using log rank test among groups. No imputation was performed for missing data. All tests were two sided, and the *P*-value of less than 0.05 was considered for statistical significance. Statistical analysis was performed using Stata 15.0 (College Station, TX, USA).

## Results

### Study population

**Fig. 1:**
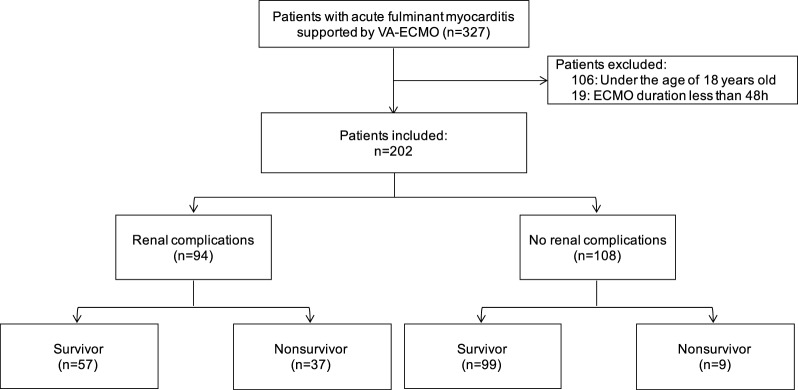
The flowchart of patients with AFM receiving VA-ECMO support. AFM acute fulminant myocarditis, VA-ECMO venoarterial extracorporeal membrane oxygenation, ECMO extracorporeal membrane oxygenation

Between January 1, 2017 and December 31, 2019, a total of 327 patients receiving VA-ECMO treatment for AFM were reported to the CSECLS database. We exclude 106 patients under the age of 18 years old and 19 patients whose ECMO duration was less than 48 h. 202 adult patients were included in the final analysis. The median age of ECMO recipients was 38 years (IQR 29–48 year) and males (*n* = 103) represented 51.0% of the total accounted patients. The duration of ECMO support was 142.9 h (IQR 112.1–188.8 h). Of 202 patients, 178 (88.1%) patients successfully weaned from ECMO. The 30-day mortality was 22.8% and the 90-day mortality was 23.8%. The main cause of death was multiple organ failure. The flow diagram of patient selection is presented in Fig. 1.

## The occurrence rate of renal complications

**Fig. 2:**
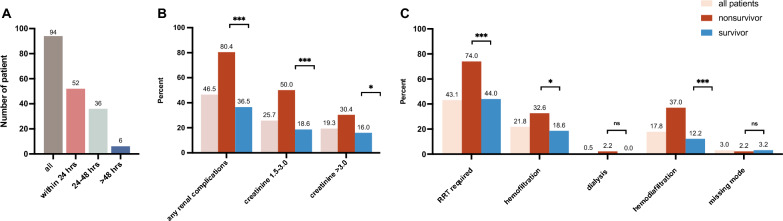
The distribution of onset time and categories of renal complications in patients with AFM supported by VA-ECMO. **A** The onset time distribution of renal complications in patients with AFM supported by VA-ECMO. **B** The distribution of serum creatinine level in patients with AFM supported by VA-ECMO. **C** The distribution of RRT mode in patients with AFM supported by VA-ECMO. AFM acute fulminant myocarditis, VA-ECMO venoarterial extracorporeal membrane oxygenation, hrs hours, RRT renal replacement therapy

High incidence of renal complications was observed in patients with AFM receiving VA-ECMO support. Of the study cohort, 46.5% (94 of 202) of patients developed renal complications according to ELSO criteria. Among these patients, 92.6% (87 of 94) needed RRT therapy (Table 1, Fig. 2). The occurrence rate of renal complications within the first 24 h on ECMO was 25.8% (52 of 202) and increased to 43.6% (88 of 202) within 48 h on ECMO, accounting for 55.3% (52 of 94) and 93.6% (88 of 94) patients with renal complications separately. The onset time of renal complications was 20(12, 45.7) hours after ECMO initiation (Fig. 2).

**Table 1:** Clinical characteristic of patients with acute fulminant myocarditis receiving VA ECMO support

Variables	All patients (*n* = 202)	Nonsurvival (*n* = 46)	Survival (*n* = 156)	*p-*value^a^
Age (years)	38 (29, 48)	39 (29, 46)	38 (29, 48)	0.830
Male gender (*n*, %)	103 (51.0)	22 (47.8)	81 (51.9)	0.625
Weight (kg)	63.3 (10.9)	63.3 (9.4)	63.3 (11.3)	0.149
Height(cm)	166.1 (7.7)	167.6 (8.3)	165.6 (7.5)	0.999
Comorbidities (*n*, %)				
History of PCI	7 (3.5)	3 (6.5)	4 (2.6)	0.196
History of myocardial infarction	2 (1.0)	0 (0)	2 (1.3)	> 0.999
NYHA class III or IV	50 (24.8)	9 (19.6)	41 (26.3)	0.261
Chronic pulmonary disease	1 (0.5)	1 (2.2)	0 (0)	0.228
Hypertension	11 (5.4)	2 (4.3)	9 (5.8)	> 0.999
Diabetes mellitus	5 (2.5)	2 (4.3)	3 (1.9)	0.320
Cerebrovascular disease	2 (1.0)	0(0)	2 (1.3)	> 0.999
Severity of illness at ECMO initiation				
SOFA	11 (7, 13)	11 (8, 15)	11 (7, 13)	0.678
SAVE	5 (0, 9)	3 (−4, 6)	7 (2, 10)	0.290
Prior^a^ cardiac arrest (*n*, %)	41 (20.3)	17 (40.0)	24 (15.4)	0.001
Prior^b^ blood pressure (mmHg)				
Systolic	72 (55, 85)	63 (43, 82)	75 (60, 85)	0.186
Diastolic	44 (31, 56)	38 (24, 56)	45 (36, 56)	0.079
Mean	53 (40, 65)	49 (30, 65)	54 (44, 65)	0.230
Prior^b^ arterial blood gas				
pH	7.33 (7.19, 7.41)	7.24 (7.12, 7.39)	7.35 (7.20, 7.41)	0.137
PaO_2_ (mmHg)	77.9 (57.8, 119.0)	79.0 (54.0, 176.0)	77.5 (57.9, 109.9)	0.289
PaCO_2_ (mmHg)	31.0 (25.6, 38.0)	34.9 (26.0, 38.0)	30.9 (30.0, 37.8)	0.546
HCO_3_^−^ (mmol/L)	18.0 (13.4, 20.4)	16.6 (11.9, 20.0)	18.0 (14.5, 20.5)	0.177
Lactate (mmol/L)	4.8 (2.5, 9.5)	6.5 (2.6, 11.8)	4.1 (2.4, 8.5)	0.139
Prior^b^ echocardiogram				
Left ventricular EF (%)^c^	30 (22, 39)	30 (21, 39)	30 (22, 38)	0.938
Left ventricular end diastolic diameter (cm)	4.5 (4.2, 5.2)	4.5 (4.2, 5.0)	4.5 (4.2, 5.2)	0.659
Prior^b^ vasopressors				
Norepinephrine (μg/kg/min)	0.6 (0.2, 1.0)	1.0 (0.3, 1.3)	0.6 (0.2, 1.0)	0.087
Epinephrine (μg/kg/min)	0.11 (0.03, 0.20)	0.20 (0.10, 0.45)	0.08 (0.02, 0.20)	0.111
VIS	60 (26, 115)	90 (45, 143)	60 (25, 100)	0.135
Prior^a^ Organ supports				
Invasive mechanical ventilation (*n*, %)	119 (58.9)	34 (73.9)	85 (54.5)	0.019
Invasive mechanical ventilation duration (hrs)	1.7(0.5, 9.5)	1.9(0.5, 11.3)	1.8(0.5, 9.0)	0.684
IABP (*n*, %)	37 (18.3)	9 (19.6)	28 (17.9)	0.803
Indication				0.004
Circulatory shock	184 (91.1)	37 (80.4)	147 (94.2)	
ECPR	18 (8.9)	9 (19.6)	9 (5.8)	
ECMO flow at 24 h (L/min)^d^	3.2 (2.9, 3.7)	3.2 (2.8, 3.8)	3.2 (2.9, 3.5)	0.887
SOFA score at 24 h	8 (6,13)	9 (7, 15)	7 (5,12)	0.496
Arterial blood gas at 24 h				
pH	7.42 (7.36, 7.48)	7.36 (7.29, 7.43)	7.44 (7.38, 7.49)	0.001
PaO_2_ (mmHg)	141.5 (97.0, 218.5)	131.5 (93.0, 284.9)	145.0 (99.0, 205.5)	0.465
PaCO_2_ (mmHg)^e^	37.0 (31.7, 41.3)	39.0(33.5, 42.9)	36.1 (31.0, 40.3)	0.129
HCO_3_^−^ (mmol/L)	24.0 (21.0, 28.0)	22.0 (18.0, 27.2)	24.8 (22.1, 28.2)	0.015
Lactate (mmol/L)	2.4 (1.5, 4.2)	3.9 (2.6, 10.4)	2.0 (1.3, 3.4)	< 0.001
Vasopressors at 24 h				
Norepinephrine (μg/kg/min)^f^	0.2 (0.1, 0.7)	0.6 (0.2, 1.0)	0.2 (0.1, 0.5)	0.006
Epinephrine (μg/kg/min)	0.05 (0.02, .10)	0.08 (0.05, 0.15)	0.04 (0.02, 0.10)	0.106
VIS	20 (8, 69)	51 (12, 100)	18 (6, 50)	0.043
Any renal complications (n, %)	94 (46.5)	37 (80.4)	57 (36.5)^g^	< 0.001
Elevated creatinine				
Creatinine 1.5–3.0 mg/dl	52 (25.7)	23 (50.0)	29 (18.6)	< 0.001
Creatinine > 3.0 mg/dl	39 (19.3)	14 (30.4)	25 (16.0)	0.011
RRT required				
Hemofiltration required	44 (21.8)	15 (32.6)	29 (18.6)	0.043
Dialysis required	1 (0.5)	1 (2.2)	0 (0)	0.228
Hemodiafiltration	36 (17.8)	17 (37.0)	19 (12.2)	< 0.001
Receiving RRT with missing mode	6 (3.0)	1 (2.2)	5 (3.2)	> 0.999
ECMO duration (hrs)	142.9 (112.1, 188.8)	148.3 (99.4, 216.0)	142.9 (113.8, 185.6)	< 0.001
Successful weaning of ECMO (*n*, %)	178 (88.1)	22 (47.8)	156 (100)	< 0.001
Invasive mechanical ventilation duration (days)	7.0 (5.0, 10.0)	7.5 (4.9, 11.1)	7.0 (5.2, 9.2)	0.339
Length of ICU stay (days)	13 (8, 20)	8 (5, 12)	14 (9, 20)	< 0.001
Length of hospital stay (days)	18 (12, 28)	8 (5, 12)	20 (14, 29)	< 0.001

*PCI* Percutaneous coronary intervention, *NYHA* New York Heart Association, *ECMO* Extracorporeal membrane oxygenation, *SOFA* Sequential Organ Failure Assessment, *SAVE* Survival after veno-arterial-ECMO, *PaO*_*2*_ Partial pressure of arterial oxygen, *PaCO*_*2*_ Partial pressure of arterial carbon dioxide, *HCO3*^*−*^ Bicarbonate, *VIS* Vasoactive-inotropic score, *IABP* Intra-aortic balloon pump, *ECPR* Extracorporeal cardiopulmonary resuscitation, *RRT* Renal replacement therapy

^a^The *p*-value represents the result of comparison of nonsurvial group and survival group

^b^Prior to ECMO initiation

^c^EF was assessed before cardiac arrest or ECMO initiation

^d^Missing values: 3%

^e^Missing values: 5%

^f^Missing values: 9%

^g^Among survivors, 3 patients receiving RRT didn’t acquired elevated creatinine

## Patient outcome

Cardiac arrest prior to ECMO (40.0% vs 15.4%; *P* = 0.001) and receiving invasive mechanical ventilation before ECMO initiation (73.9% vs 54.5%) was more obviously commonly observed in non-survivors compared with survivors. There was no significant difference of SOFA score, systolic blood pressure (63(43, 82) vs 75(60, 85) mmHg; *P* = 0.186), diastolic blood pressure [38(24, 56) vs 45(36, 56) mmHg, *P* = 0.079] lactate concentration [6.5(2.6, 11.8) vs 4.1(2.4, 8.5); *P* = 0.139] and VIS before initiation between two groups (Table 1).

At 24 h after ECMO initiation, ECMO blood flow and SOFA score was similar between two groups. Compared with survivors, nonsurvivors had lower pH value [7.36 (7.29, 7.43) vs 7.44 (7.38, 7.49), *P* = 0.001], bicarbonate concentration [22.0 (18.0, 27.2) vs 24.8 (22.1, 28.2) mmol/L, *P* = 0.015], higher lactate concentration [3.9 (2.6, 10.4) vs 2.0 (1.3, 3.4) mmol/L, *P* < 0.001], higher VIS [51 (12, 100) vs 18 (6, 50), *P* = 0.043] and longer ECMO duration[148.3 (99.4, 216.0) vs 142.9 (113.8, 185.6) hours, *P* < 0.001]. There was no significant difference of invasive mechanical ventilation duration between two groups (Table 1).

## Risk factors of renal complications

**Fig. 3:**
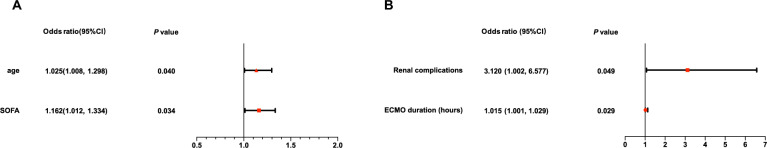
Adjusted odds ratios of renal complications. Forrest plot of risk-adjusted odds ratios of renal complications (**A**) and 30-day mortality (**B**). ECMO extracorporeal membrane oxygenation

Potential risk factors of renal complications by univariable analysis include age, SOFA score, cardiac arrest, receiving invasive mechanical ventilation, receiving IABP, MAP, pH value, lactate concentration and VIS prior to ECMO initiation (Table 2). Multivariable logistic regression analysis identified that age (Odds ratio (OR) 1.025, 95% Confidence Interval (CI) 1.008–1.298, *P* = 0.040) and SOFA score (OR 1.162, 95%CI 1.012–1.334, *P* = 0.034) were independent risk factors of renal complications developing in patients with AFM receiving VA-ECMO (Fig. 3A). The AUROC of the logistic model was 0.761(95%CI 0.621–0.870) (Additional file 2: Fig. S1). Hosmer–Lemeshow test *P*-value was 0.345.

**Table 2:** Logistic regression analysis of potential risk factors associated with renal complications

Variables	Univariable analysis		Multivariable analysis	
	Unadjusted OR (95% CI)	*p-*value	Adjusted OR (95% CI)	*p-*value
Age	1.317 (1.256, 1.416)	0.048	1.025 (1.008, 1.298)	0.040
Gender	1.089 (0.626, 1.893)	0.748		
SOFA score	1.128 (1.086, 1.399)	0.048	1.162 (1.012, 1.334)	0.034
Prior^a^ ECMO cardiac arrest	4.822 (2.211, 10.518)	< 0.001		
Prior^a^ MV^b^	3.202 (1.768, 5.797)	< 0.001		
Prior^a^ IABP^b^	1.894 (0.917, 3.911)	0.084		
Prior^a^ MAP (mmHg)	0.975 (0.959, 0.991)	0.003		
Prior^a^ pH value	0.124 (0.010, 1.464)	0.097		
Prior^a^ lactate concentration (mmol/L)	1.148 (1.063, 1.239)	< 0.001		
Prior^a^ VIS	1.006 (1.003, 1.011)	0.029		

*SOFA* Sequential Organ Failure Assessment, *ECMO* extracorporeal membrane oxygenation, *MV* mechanical ventilation, *IABP* intra-aortic balloon pump, *MAP* mean arterial pressure, *VIS* vasoactive-inotropic score

^a^Prior to ECMO initiation

^b^Analyzed as categorical variables

## Impact of renal complications on patient mortality

**Fig. 4:**
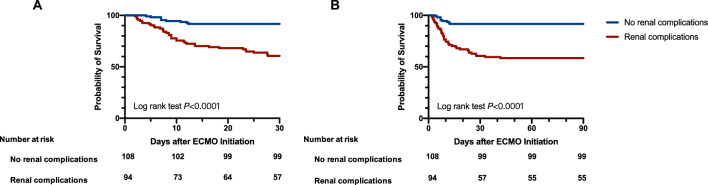
**A**. Kaplan–Meier curves of cumulative probabilities of 30-day survival for patients with AFM supported by VA-ECMO concomitant with renal complications or not. **B**. Kaplan–Meier curves of cumulative probabilities of 30-day survival for patients with AFM supported by VA-ECMO concomitant with renal complications or not. AFM acute fulminant myocarditis, VA-ECMO venoarterial extracorporeal membrane oxygenation

A significantly higher 30-day mortality rate in patients with renal complications (39.4%) was observed compared with patients without renal complications (4.5%) (Table 1). Kaplan–Meier survival curves differed significantly for patients with renal complications versus patients without renal complications (Log rank test *P* < 0.0001) (Fig. 4A, 30-day mortality; Fig. 4B 90-day mortality). After adjusting potential prognostic factors of 30-day mortality including cardiac arrest, MAP, SOFA score, pH value, lactate concentration and VIS prior to ECMO, renal complications (OR 3.120, 95%CI 1.002–6.577, *P* = 0.049) was related to 30-day mortality (Table 3) (Fig. 3B). ECMO duration (OR 1.015, 95%CI 1.001–1.029, *P* = 0.029) was another independent risk factor of 30-day mortality. The AUROC of the logistic model was 0.818(95%CI 0.673–0.954). Hosmer–Lemeshow test *P*-value was 0.203(Additional file 2, Fig. S2).

**Table 3:** Logistic regression analysis of potential risk factors associated with 30-day mortality

Variables	Univariable analysis		Multivariable analysis	
	Unadjusted OR (95% CI)	*p-*value	Adjusted OR (95% CI)	*p-*value
Age	0.992 (0.967, 1.018)	0.555		
Gender	0.848 (0.439, 1.639)	0.625		
Prior^a^ ECMO cardiac arrest	3.224 (1.538, 6.757)	0.002		
Prior^a^ MAP (mmHg)	0.985 (0.969, 1.001)	0.071		
Prior^a^ SOFA score	1.224 (1.163, 1.921)	0.045		
Prior^a^ pH value	0.040 (0.003, 0.558)	0.017		
Prior^a^ lactate concentration (mmol/L)	1.076 (1.001, 1.157)	0.046		
Prior^a^ VIS	1.005 (1.001, 1.010)	0.020		
ECMO duration (hours)	1.020 (1.012, 1.030)	0.079	1.015 (1.001, 1.029)	0.029
Renal complications	7.140 (3.215, 15.859)	< 0.001	3.120 (1.002, 6.577)	0.049

*SOFA* Sequential Organ Failure Assessment, *ECMO* extracorporeal membrane oxygenation, *MV* mechanical ventilation, *IABP* intra-aortic balloon pump, *MAP* mean arterial pressure, *VIS* vasoactive-inotropic score

^a^Prior to ECMO initiation

^b^Analyzed as categorical variables

## Discussion

This is a national-level study to evaluate the impact of renal complications on outcome and risk factors for developing renal complications in adult patients with AFM receiving VA-ECMO in China. In this population, the occurrence rate of renal complications was up to 46.5% and independently associated with 30-day mortality. Age and SOFA score prior to ECMO initiation were related to the development of renal complications.

Patients with AFM receiving VA-ECMO at high risk of developing renal complications. The reported incidence of renal complications in patients with myocarditis receiving VA-ECMO support ranged from 17.5% to 55.8% [30–33]. The occurrence rate of renal complications in our cohort was 46.5%. Differences in patient characteristics, clinical practice and criteria for renal dysfunction contribute to disparity in the incidence of renal complications. The underlying mechanisms of renal complications in patients with AFM receiving VA-ECMO support are complicated and multifactorial including patient-related factors, MV related factors, ECMO-related factors and so on. Prior to ECMO initiation, hemodynamic instability, low cardiac output [14, 34], hypoxemia [35], inflammatory cytokines [36] and other illness-related factors [37, 38], alone or in combination, contribute to renal dysfunction. MV is widely used in patients with cardiogenic shock for the management of acute hypoxemia, increased work of breathing, airway protection, and hemodynamic or electric instability [39]. Positive end-expiratory pressure could reduce work of and promote recovery of myocardium by decreasing venous return and left ventricular pre-load. It’s Deterioration of cardiac function was related to delays in MV initiation, which might contribute to disorders of renal function. Diepen et al. reported that each one hour delay in intubation from the myocardial injury onset was independently associated with mortality [8, 40]. Following ECMO initiation, continuous flow [41], ischemia–reperfusion [35] and blood exposure to artificial surfaces which may lead to elevated production of reactive oxygen species [42] and hemolysis [43], may increase risk of renal dysfunction.

The results of this study magnify that renal complications are in relation with worse outcomes of patients with AFM receiving VA-ECMO. Previous study demonstrated the negative prognostic effect of renal dysfunction on patients with circulatory shock supported by VA-ECMO [31, 44]. Renal dysfunction has adverse effects on remote organs included heart through various pathways including inflammation [45], metabolic and hemodynamic alterations [46], and the neurohormonal system [7, 47]. Among physiological disturbances after renal dysfunction, fluid overload is absolutely important as it increases cardiac preload and stretches cardiomyocytes, which lead to diminishing contractility and increasing work demand. Renal dysfunction-based accumulated acid damage the myocardium through altered expression of β-receptors and mishandling of intracellular calcium [48]. Electrolyte disorders induced by renal dysfunction may lead to cardiac arrhythmias which contributes to decreased cardiac output, hemodynamic instability and increased risk of thrombotic events [49]. Furthermore, increased activity of the renin–angiotensin–aldosterone and central nervous systems may cause increased fluid retention, increasing pre- and afterload [46]. When the kidney hurts, other organs also suffer contributing to the renal dysfunction-associated mortality and morbidity [7, 50, 51]. In our study, the development of renal complications was associated with 3.12-fold increase risk of 30-day mortality. It’s definitely important to prevent and treat renal complications in patients receiving VA-ECMO support and avoid accompanied complications or further multiple organ dysfunction.

Our study demonstrated that advancing age was an independent risk factor of renal complications with one year increase in age increasing the odds of renal complications by 2.5%. Lorusso et al. demonstrated that elderly patients (≥ 70 years old) had a higher rate of multiorgan failure and was an independent predictor (adjusted OR 1.043, 95%CI 1.023–1.064, *P* < 0.001) of in-hospital mortality through analyzing the data from ELSO [52]. A recent meta-analysis by Mou et al. showed that increasing age was related with higher mortality [13]. As recent studies pointed out, kidney undergone a series of transcriptomic, hemodynamic and physiologic changes which could impair the ability of the kidney to withstand and recover from injury. Multiple pathways interact to produce these changes including increasing oxidative stress [53, 54], increasing angiotensin II [55], decreasing peroxisome proliferator–activated receptor-*γ* levels [56] and many other complex ones [57].

Furthermore, our study verified that SOFA score was associated with renal complications. Antonucci et al. reported that patients with AKI receiving ECMO had higher SOFA score [58]. A recent meta-analysis demonstrated that severe acute kidney injury was related to higher SOFA score, diabetes mellitus and longer duration of ECMO support [13]. The dysfunction of one organ is communicated to the impaired function of other organs via complex pathways [47, 59–61]. The SOFA score has widely employed in the assessment of organ dysfunction. Patients with higher SOFA score had greater number and (or) severity of dysfunctional organ, which might have more negative effect on renal function [38].

Results obtained in our study should be tempered by several limitations. Firstly, this was a retrospective study which demonstrated an association but not the casual relationship between risk factors and outcomes. Secondly, the severity of renal complications was mainly evaluated based on serum creatinine level while data on urine output were not collected by the database, which limited comprehensive assessment of patient renal function and exploration on the timing RRT initiation for patients with AFM receiving ECMO support. Third, some important detailed data were lacking on numerical serum creatinine level and urine output before ECMO initiation and while on ECMO, daily fluid balance, the time points when serum creatinine levels peaked, transfusion prior to and on ECMO support, the class and dose of nephrotoxic drugs, hypoxia exposure and other factors. Prospective study including all those parameters was needed to confirm our findings. Fourth, data to define the link between ECMO and renal complications was insufficient, further research are needed about the underlying pathophysiological mechanisms. Fifth, patients are solely identified as having AFM by clinical criteria in the current study. EMB or MRI scans were not performed on any patients to confirm the diagnosis. This exposes potential biases in the results and misdiagnoses. Finally, data on the recovery of renal function were not collected in database, we could not assess the relation between renal complications while on ECMO and long-term renal function.

## Conclusion

In our study, patients with AFM receiving VA ECMO at high risk of developing renal complications. Age and SOFA score was associated with increased risk of developing renal complications. The onset of renal complications contributed to 30-day mortality.

### Supplementary Information

**Additional file 1**. STROBE statement.

**Additional file 2:** **Table S1** Diagnostic criteria for clinical suspected myocarditis. **Figure S1** Receiver operating characteristic curve calculated for multivariable logistic regression of renal complications. Figure S2 Receiver operating characteristic curve calculated for multivariate logistic regression of 30-day mortality.

## Data Availability

The datasets used and analyzed during the current study are available from the corresponding author upon reasonable request, but not publicly available.

## Abbreviations

AFM: Acute fulminant myocarditis
ECMO: Extracorporeal membrane oxygenation
VA-ECMO: Venoarterial extracorporeal membrane oxygenation
VV-ECMO: Venovenous extracorporeal membrane oxygenation
CSECLS: Chinese Society of ExtraCorporeal Life Support
STROBE: STrengthening the Reporting of OBservational studies in Epidemiology
SOFA: Sequential Organ Failure Assessment
SAVE: Survival after veno-arterial-ECMO
ECPR: Extracorporeal cardiopulmonary resuscitation
EF: Ejection fraction
VIS: Vasoactive-inotropic score
IMV: Invasive mechanical ventilation
ICU: Intensive care unit
ELSO: Extracorporeal Life Support Organization
RRT: Renal replacement therapy
IQR: Interquartile range; AUROC, area under the receiver operating curve
IABP: Intra-aortic balloon pump
MAP: Mean arterial pressure
OR: Odds ratio
CI: Confidence interval
