# Supplementary material for: Impact of renal complications on outcome in adult patients with acute fulminant myocarditis receiving venoarterial extracorporeal membrane oxygenation: an analysis of nationwide CSECLS database in China
Source: Ann Intensive Care. 2023 Sep 27;13:93. doi: 10.1186/s13613-023-01186-x (PMC10533475; doi:10.1186/s13613-023-01186-x)
Supplement: Supplementary file 2 — Additional file 2: Table S1 Diagnostic criteria for clinical suspected myocarditis. Figure S1 Receiver operating characteristic curve calculated for multivariable logistic regression of renal complications. Figure S2 Receiver operating characteristic curve calculated for multivariate logistic regression of 30-day mortality. [file 13613_2023_1186_MOESM2_ESM.docx]

**Additional file 2**

**Impact of Renal Complications on Outcome in Adult Patients with Acute Fulminant Myocarditis Receiving Venoarterial Extracorporeal Membrane Oxygenation: an Analysis of Nationwide CSECLS Database in China**

**Tables of Contents of the Additional file 2:**

1. **Table S1** Diagnostic criteria for clinical suspected myocarditis.

2. **Fig. S1** Receiver operating characteristic curve calculated for multivariable logistic regression of renal complications.

3. **Fig. S2** Receiver operating characteristic curve calculated for multivariate logistic regression of 30-day mortality.

**Table S1 Diagnostic criteria for clinical suspected myocarditis**

| **Clinical presentations** | Acute chest pain, pericarditic, or pseudo-ischaemic |
| --- | --- |
|  | New-onset (days up to 3 months) or worsening of dyspnoea at rest or exercise, and/or fatigue, with or without left and/or right heart failure signs |
|  | Palpitation, and/or unexplained arrhythmia symptoms and/or syncope, and/or aborted sudden cardiac death |
|  | Unexplained cardiogenic shock |
| **Diagnostic criteria** | ECG/Holter/stress test features:  Newly abnormal 12 lead ECG and/or Holter and/or stress testing, any of the following: I to III degree atrioventricular block, or bundle branch block, ST/T wave change (ST elevation or non ST elevation, T wave inversion), sinus arrest, ventricular tachycardia or fibrillation and asystole, atrial fibrillation, reduced R wave height, intraventricular conduction delay (widened QRS complex), abnormal Q waves, low voltage, frequent premature beats, supraventricular tachycardia |
|  | Myocardiocytolysis markers: Elevated TnT/TnI |
|  | Functional and structural abnormalities on cardiac imaging (echo/angio/CMR):  New, otherwise unexplained LV and/or RV structure and function abnormality (including incidental finding in apparently asymptomatic subjects): regional wall motion or global systolic or diastolic function abnormality, with or without ventricular dilatation, with or without increased wall thickness, with or without pericardial effusion, with or without endocavitary thrombi |
|  | Tissue characterization by CMR, at least two of the following criteria present:   1. Regional or global myocardial signal intensity increase in T2-weighted oedema images 2. Increased global myocardial early gadolinium enhancement ratio between myocardium and skeletal muscle in gadolinium-enhanced T1-weighted images 3. There is at least one focal lesion with non-ischemic regional distribution in inversion recovery-prepared gadolinium-enhanced T1-weighted images (late gadolinium enhancement) |

^*^ Acute myocarditis was clinically suspected if patients had more than one clinical presentation and more than one diagnostic criteria from different categories in the absence of any known pre-existing cardiovascular disease or extra-cardiac causes that could explain the syndrome. If the patients were asymptomatic, more than two diagnostic criteria should be met. In all suspected myocarditis, coronary angiography was performed for excluding the diagnosis of coronary artery disease (coronary stenosis ≥ 50%).

* Acute fulminant myocarditis (AFM) was defined as patients with acute myocarditis who needed inotropic drugs and/or mechanical circulatory support including ECMO to maintain end organ perfusion.

**Fig. S1** Receiver operating characteristic curve calculated for multivariable logistic regression of renal complications. AUROC area under the receiver operating curve.

**Fig. S2** Receiver operating characteristic curve calculated for multivariate logistic regression of 30-day mortality. AUROC area under the receiver operating curve.
